# Preoperative Prediction of Fat-Suppressed T2-Weighted Imaging-based Breast Edema Scores for Lymphovascular Invasion in Patients with Breast Cancer

**DOI:** 10.2174/0115734056420967260115075602

**Published:** 2026-02-03

**Authors:** Ruihong Sun, Xi Wang, Yun Hu, Zengfa Huang, Hongyuan Liu, Xiang Wang

**Affiliations:** 1 Department of Radiology, Central Hospital of Wuhan, Tongji Medical College, Huazhong University of Science and Technology, Wuhan 430014, China; 2 Cancer Center, Union Hospital, Tongji Medical College, Huazhong University of Science and Technology, Wuhan 430022, China; 3 Hubei Key Laboratory of Precision Radiation Oncology, Wuhan 430022, China; 4 Institute of Radiation Oncology, Union Hospital, Tongji Medical College, Huazhong University of Science and Technology, Wuhan 430022, China

**Keywords:** Breast cancer, Edema, Breast edema score, Magnetic resonance imaging, Fat-suppressed T2-weighted imaging, Lymphovascular invasion

## Abstract

**Introduction:**

Lymphovascular invasion (LVI) is a critical prognostic factor in breast cancer, typically diagnosed *via* postoperative pathology. This study aimed to evaluate whether fat-suppressed T2-weighted imaging (FS T2WI)-based breast edema score (BES) combined with clinicopathological features could preoperatively predict LVI status.

**Materials and Methods:**

This retrospective study enrolled 574 breast cancer patients who underwent MRI and surgery from January 2021 to December 2023. Patients were classified as LVI-positive (n=174) or LVI-negative (n=400) based on postoperative pathology. Breast edema on FS T2WI was scored from 1 to 4 (BES 1, no edema; BES 2, peritumoral edema; BES 3, prepectoral edema; and BES 4, subcutaneous edema). Univariate and multivariate binary logistic regression analyses were performed to identify risk factors associated with LVI. A clinicopathological model and a combined BES–clinicopathological model were constructed, and diagnostic performance was evaluated using the area under the receiver operating characteristic curve (AUC) and the DeLong test.

**Results:**

Multivariate analysis revealed that BES and clinicopathological factors, including age, axillary lymph node metastasis, and tumor size, were independent predictors of LVI. Compared with BES 1, tumors with BES 2, BES 3, and BES 4 were associated with a 1.825-, 2.047-, and 4.341-fold increased LVI risk, respectively. The combined BES–clinicopathological model outperformed the clinicopathological model alone (AUC, 0.765 *vs*. 0.778; *P*<0.05).

**Discussion:**

Higher BES was independently associated with increased LVI risk. The predictive model integrating BES with clinicopathological variables outperformed single-parameter models, suggesting that BES may provide complementary imaging biomarkers for assessing tumor aggressiveness. Validation in larger, multicenter cohorts is warranted.

**Conclusion:**

FS T2WI-based BES combined with clinicopathological features may improve preoperative prediction of LVI in breast cancer and support individualized treatment planning.

## 1 INTRODUCTION

Breast cancer is the most common malignancy among women worldwide, with postoperative recurrence and metastasis being the leading causes of treatment failure and mortality [1]. As a key pathological feature driving metastasis, lymphovascular invasion (LVI), defined as infiltration of cancer cells into stromal capillaries and lymphatic vessels, followed by vascular dissemination, strongly correlates with increased risks of local recurrence, axillary lymph node (ALN), distant metastasis, and poor prognosis in patients with breast cancer [2-5]. Accurate preoperative assessment of LVI status is therefore critical for guiding clinical decisions, such as optimizing surgical margin and selecting neoadjuvant therapy strategies [5]. However, current LVI diagnosis relies on invasive biopsy or postoperative histopathology, lacking timely postoperative data for personalized treatment. This clinical limitation demands urgent, noninvasive preoperative LVI-prediction tools.

Both histopathological examination and magnetic resonance imaging (MRI) provide essential information for evaluating the biological behavior and detection of breast cancer. Histopathology reveals tumor cellular and molecular characteristics, whereas MRI offers a noninvasive means to assess tumor morphology and microenvironmental changes, making the two methods complementary in clinical assessment. Previous studies have shown that MRI features, such as peritumoral edema, the apparent diffusion coefficient (ADC), and adjacent vessel signs, are associated with LVI [6, 7]. However, their predictive value has not been fully established, and most research has focused primarily on peritumoral edema, neglecting broader patterns of breast edema. Focal breast edema (FBE), which includes peritumoral, prepectoral, and subcutaneous edema types [8], has recently gained attention as a potential biomarker of tumor aggressiveness. The breast edema score (BES), introduced in 2021 to categorize FBE on T2-weighted imaging (T2WI) [9], was subsequently shown to predict sentinel lymph node metastasis [10-12] and to assess response to neoadjuvant therapy [13, 14], supporting its utility in assessing tumor biology. Despite evidence that breast edema is associated with tumor aggressiveness and prognosis, whether BES on fat-suppressed (FS) T2WI can serve as an imaging marker for predicting LVI, beyond peritumoral edema alone, has yet to be systematically evaluated.

This study aimed to assess whether preoperative T2WI-based BES of FS could predict LVI in breast cancer and to develop a model combining clinicopathological features for individualized treatment planning.

## 2 MATERIALS AND METHODS

### 2.1 Patients

This retrospective study was approved by our Institutional Review Board and Ethical Committee, with a waiver of informed consent. Consecutive patients who underwent breast MRI at our hospital from January 2021 to December 2023 were included according to the following inclusion criteria: (1) breast cancer confirmed by histopathology; (2) pathologically confirmed LVI status; and (3) preoperative MRI performed within two weeks before surgery. The exclusion criteria were as follows: (1) biopsy before MRI; (2) prior treatment before MRI; (3) neoadjuvant chemotherapy; (4) inadequate MRI image quality; and (5) incomplete clinical or pathological data. A total of 574 lesions from 574 patients were included. The patient selection process is illustrated in Fig. (**1**).

### 2.2 MRI Technique

Breast MRI examinations were performed on a 3.0T scanner (Skyra, Siemens Healthcare) with a 16-channel breast coil and patients in the prone position. The parameters for the MR scans were as follows: (1) axial T1-weighted imaging (T1WI): repetition time (TR), 735 ms; echo time (TE), 8.1 ms; field of view (FOV), 320 mm × 320 mm; matrix, 240 × 320; and slice thickness, 4.0 mm; (2) axial FS-T2WI *via* Dixon fat suppression technology: TR, 3700 ms; TE, 101 ms; FOV, 320 mm × 320 mm; matrix, 224 × 320; and slice thickness, 4.0 mm; (3) axial diffusion-weighted imaging (DWI) with multiple b values (0, 50, 1000, 2000 s/mm^2^): TR, 5000 ms; TE, 70 ms; FOV, 280 mm × 170 mm; matrix, 114 × 188; and slice thickness, 5.0 mm; and (4) axial dynamic contrast-enhanced MRI (DCE-MRI) using a T1-weighted time-resolved interleaved acquisition sequence: TR, 5.24 ms; TE, 2.46 ms; FOV, 320 mm × 260 mm; matrix, 182 × 320; slice thickness, 1.5 mm; flip angle, 10°. Gadopentetate dimeglumine (0.1 mmol/kg) was injected at 2 mL/s, followed by a 20 mL saline flush. Seven consecutive dynamic phases were acquired, each lasting 50 seconds.

### 2.3 Clinical-Pathologic Evaluation

Clinical data (age, menstrual status, family history) were obtained from electronic medical records. Pathological variables included histological type, estrogen receptor (ER) status, progesterone receptor (PR) status, human epidermal growth factor receptor 2 (HER2) status, Ki-67 index, ALN status, and LVI status. LVI was defined as clustered cancer cells within small blood or lymphatic vessels in the peritumoral area. ER/PR positivity was defined as ≥1% nuclear staining. HER2 positivity was defined as an immunohistochemistry score of 3+ or a score of 2+ with confirmed gene amplification by fluorescence in situ hybridization (FISH) [15]. The Ki-67 index was defined using a cutoff of 14%. Breast cancer subtypes were classified according to the 2013 St. Gallen consensus: luminal A, luminal B, HER2-enriched, and triple-negative [16].

### 2.4 MRI Analysis

MRI features were independently reviewed by two radiologists with 7 and 10 years of experience, blinded to clinical data. Discrepancies were resolved by a senior radiologist (>15 years of experience). MRI features assessed per Breast Imaging Reporting and Data System (BI-RADS) included: (1) fibroglandular tissue (FGT) density (fatty, scattered fibroglandular, heterogeneous dense, or extremely dense); (2) background parenchymal enhancement (BPE) (minimal, mild, moderate, or marked); (3) tumor laterality (right or left breast); (4) tumor size, measured as the maximum diameter on the second phase of DCE imaging; (5) lesion type (mass or nonmass enhancement); (6) mass shape (round/oval or irregular); (7) mass margin (circumscribed or noncircumscribed); (8) internal enhancement pattern of the mass (homogeneous, heterogeneous, or rim enhancement); and (9) time‒intensity curve (TIC) type (persistent, plateau, or washout).

FBE on FS T2WI was scored independently by two radiologists, blinded to clinical data. Disagreements were resolved *via* consensus with a senior radiologist. FBE was categorized into four BES grades based on prior studies [8, 9, 17, 18]. BES 1 (no edema) was defined as the absence of a visible edema signal on FS T2WI (Fig. **2a**). BES 2 (peritumoral edema) referred to the presence of linear or localized areas of waterlike high signal intensity adjacent to the tumor (Fig. **2b**); BES 3 (prepectoral edema) was characterized by linear or bandlike high signal intensity extending into the space anterior to the pectoralis major muscle (Fig. **2c**); and BES 4 (subcutaneous edema) was defined as prominent high signal intensity within the skin and subcutaneous fat, excluding localized signal changes caused by direct tumor invasion of the skin (Fig. **2d**).

### 2.5 Statistical Analysis

Statistical analyses were performed using SPSS version 22.0 and MedCalc version 19.6. Interobserver agreement was assessed using Cohen’s kappa and weighted kappa. Normality was evaluated *via* the Kolmogorov–Smirnov test. Continuous variables are expressed as mean ± standard deviation and compared using a *t*-test. Categorical variables were compared using chi-square or Fisher’s exact tests. Variables with *P* < 0.05 in univariate analysis were included in multivariate logistic regression. Diagnostic performance was evaluated using receiver operating characteristic (ROC) curves, area under the receiver operating characteristic curve (AUC), sensitivity, and specificity. Model calibration was assessed by the Hosmer–Lemeshow test, and AUCs were compared using the DeLong test. A *P* value < 0.05 was considered statistically significant.

## 3 RESULTS

### 3.1 Interobserver Agreement Analysis

The κ coefficients for tumor size, lesion location, lesion type, mass shape, mass margin, and peritumoral edema were 0.845, 0.991, 0.892, 0.823, 0.787, and 0.855, respectively. Weighted κ coefficients for breast parenchymal type, BPE, internal enhancement pattern, TIC curve type, and BES were 0.871, 0.822, 0.784, 0.863, and 0.819, respectively. These results indicate substantial to almost perfect interobserver agreement for all MRI features.

### 3.2 Comparison of Clinical, Pathological, and MRI Characteristics

A total of 574 female patients were included (mean age, 57.1 ± 11.2 years; range, 28–81 years), of whom 174 were LVI-positive. Clinical, pathological, and MRI characteristics are summarized in Table **1**. Compared with LVI-negative patients, LVI-positive patients were younger, had higher rates of ALN positivity, higher Ki-67 index, larger tumor size, and greater prevalence of peritumoral, prepectoral, and subcutaneous edema (*P* < 0.05). No significant differences were observed between groups regarding menstrual status, family history, histological type, HER2 status, BPE, lesion laterality, lesion type, mass shape, margin, internal enhancement pattern, or TIC curve type (*P* > 0.05).

### 3.3 Univariate and Multivariate Analysis

Six variables significant in the univariate analysis, including age, ALN status, molecular subtype, Ki-67 index, maximum tumor diameter, and BES, were entered into multivariate logistic regression. Younger age (OR = 0.973, 95% CI: 0.956–0.992, *P* = 0.004), ALN positivity (OR = 2.316, 95% CI: 1.455–3.685, *P* < 0.001), a tumor size > 2 cm (OR = 3.618, 95% CI: 2.337–5.601, *P* < 0.001), and higher BES were identified as independent risk factors for LVI. Compared with BES 1, the odds ratios for LVI were 1.825 (95% CI: 1.085–3.069, *P* = 0.023) for BES 2, 2.047 (95% CI: 1.051–3.987, *P* = 0.035) for BES 3, and 4.341 (95% CI: 2.128–8.856, *P* < 0.001) for BES 4. Detailed results are presented in Table **2**.

### 3.4 Predictive Model Assessment

ROC curves and corresponding analyses are presented in Fig. (**3**) and Table **3**. The combined BES–clinicopathological model demonstrated the highest predictive performance for LVI, with an AUC of 0.778 (95% CI: 0.742–0.811), significantly higher than that the clinicopathological model alone (AUC = 0.762, 95% CI: 0.725–0.796; Z = 1.983, *P* < 0.05) and the BES-only model (AUC = 0.678, 95% CI: 0.638–0.716; Z=5.200, *P*<0.05). Its AUC was also higher than that of the FBE–clinicopathological model (AUC = 0.765, 95% CI: 0.728–0.799), although the difference was not statistically significant (Z = 1.637, *P* = 0.102). Among all models, the BES–clinicopathological model had the highest specificity (73.50%) and sensitivity (71.84%). The Hosmer–Lemeshow goodness-of-fit test indicated good calibration (*P* = 0.345).

## 4 DISCUSSION

LVI refers to the presence of tumor emboli within lymphatic or vascular structures surrounding breast cancer. Its occurrence indicates potential systemic micrometastasis, suggesting that systemic therapy may be particularly important for these patients. Therefore, LVI status plays a key role in guiding breast cancer treatment strategies [19]. Most previous studies examining the relationship between breast edema and LVI have focused mainly on peritumoral edema. In contrast, this study further evaluated prepectoral and subcutaneous edema and developed a predictive model for LVI based on BES. To our knowledge, similar studies are rare.

Our study demonstrated that the mean age of LVI-positive patients was lower than that of LVI-negative patients, indicating that younger women are at higher risk of LVI. Age was identified as an independent risk factor, consistent with the findings reported by Zhang *et al*. [20] and Houvenaeghel *et al*. [21], who also reported younger age as a high-risk factor for LVI in invasive breast cancer. Early-onset breast cancer, influenced by elevated estrogen and progesterone levels, often presents at a more advanced stage and is more likely to be associated with LVI [22]. However, no significant difference in family history of breast cancer was found, which is in line with previous reports [19-21, 23].

Univariate logistic regression analysis revealed that a higher Ki-67 index was a significant predictor of LVI in breast cancer, indicating that LVI-positive tumors tend to grow more rapidly and exhibit greater aggressiveness. Ki-67 is a widely established marker of tumor proliferation, metastasis, and prognosis, and previous studies have reported a positive association between Ki-67 expression and LVI [19, 23], consistent with our findings. In contrast, no significant correlation was observed between HER2 status and LVI, although some studies have suggested HER2 overexpression as a potential risk factor [24, 25]. LVI is a key pathway for lymph node metastasis. Our results showed that ALN positivity was an independent risk factor for LVI, indicating that tumors with nodal metastasis are more likely to exhibit LVI, in agreement with prior reports [26, 27]. Therefore, accurate preoperative imaging evaluation of the axillary lymph node may aid in predicting LVI.

Our study confirmed that the maximum tumor diameter in LVI-positive patients was significantly larger than in LVI-negative patients. Using 20 mm as a threshold, tumor size emerged as a strong independent predictor of LVI, consistent with previous studies [19, 24, 28]. To evaluate the predictive value of preoperative MRI features, the maximum tumor diameter was measured from maximum-intensity projection images, as in prior research [29]. This may reflect the fact that larger tumors often grow more rapidly, infiltrating surrounding tissues and invading capillaries and lymphatic vessels. Irregular tumor shape and noncircumscribed margins were common in both groups, with no significant difference. This contrasts with some prior reports [20, 21] and may be due to the predominance of invasive breast cancers in our cohort, which typically disrupt adjacent tissues and present with stromal proliferation and indistinct borders. No significant differences were observed in internal enhancement patterns or TIC curve types between groups. Previous studies on DCE-MRI features and LVI have yielded inconsistent results [27, 30]. Although some reported a positive association between BPE and LVI in invasive breast cancer [20, 31], our findings did not support this, aligning with the findings reported by Cheon *et al*. [6].

Multiple studies have identified breast edema as a poor prognostic factor in malignant breast disease [6, 10, 18, 32]. In our study, FBE independently predicted LVI positivity, with tumors exhibiting edema showing a higher likelihood of LVI. Focal edema on FS T2WI may thus serve as a biomarker of tumor aggressiveness. To further clarify the relationship between edema patterns and LVI, FBE was subclassified into peritumoral edema (BES 2), prepectoral edema (BES 3), and subcutaneous edema (BES 4). Compared with tumors with BES 1 (no edema), tumors with BES 2, BES 3, and BES 4 had 1.825-, 2.047-, and 4.341-fold greater odds of LVI, respectively, indicating a progressive increase in risk with higher BES levels. Our findings align with those of Cheon *et al*. [6] and Uematsu *et al*. [18], who reported a significant association between prepectoral edema and LVI. However, other studies found no statistically significant differences in prepectoral or subcutaneous edema between LVI-positive and LVI-negative groups, despite higher frequencies in the LVI-positive group [28]. This inconsistency may result from selection bias or the limited sample sizes for prepectoral and subcutaneous edema in those studies.

Our findings demonstrated that the logistic regression model combining BES and clinicopathological variables achieved the highest AUC for predicting LVI in breast cancer, significantly outperforming the clinicopathological model alone, the BES-only model, and the focal edema model (*P* < 0.05). This combined model also showed the highest specificity among all evaluated models. These results suggest that FS T2WI-based BES provides additional biological insight into LVI, with the potential to improve assessment of tumor aggressiveness and inform clinical management.

The ACR is preparing to release the 6^th^ edition of BI-RADS, with updates presented at the Society of Breast Imaging (SBI) Symposium and the Radiological Society of North America (RSNA) Annual Meeting between 2022 and 2024. Based on accumulating evidence of its prognostic significance in breast cancer [33], peritumoral edema will be added as a new associated finding. Peritumoral edema reflects a localized tissue response and is considered the mildest form of edema, whereas prepectoral edema is often associated with overt LVI, and subcutaneous edema typically indicates more advanced lymphatic involvement [34, 35]. These anatomical patterns of edema may correspond to different stages or severities of disease progression. Despite their potential clinical relevance, prepectoral and subcutaneous edema have received limited attention, and their significance remains unclear. Our study evaluated the predictive value of various types of FBE, contributing to the interpretation of T2WI features and understanding of tumor biology, and providing supplementary imaging markers to support clinical decision-making.

## 5 STUDY LIMITATION

This study had several limitations. First, as a retrospective study, patients who underwent invasive procedures, such as core needle biopsy prior to MRI, were excluded, which may have introduced selection bias. Therefore, prospective studies are needed to validate our findings. Second, given the relatively small number of cases with prepectoral edema (18.4% *vs*. 9.8%) and subcutaneous edema (17.8% *vs*. 6.0%) in the LVI-positive and LVI-negative groups, respectively, further studies with larger multicenter cohorts are warranted. Third, assessment of FBE on T2WI is subject to observer variability. Future research will focus on applying artificial intelligence (AI) for BES assessment and developing radiomic- and deep learning–based models to improve diagnostic accuracy.

## CONCLUSION

LVI is a key factor in breast cancer metastasis. This study proposes a noninvasive approach for preoperative prediction of LVI using the BES derived from FS T2WI. Our findings demonstrate a significant correlation between BES and LVI status. Higher BES levels were associated with progressively increased LVI risk, with BES 2, 3, and 4 showing greater risk than BES 1 (no edema). An integrated model combining BES with clinicopathological features significantly outperformed the clinicopathological model alone in predicting LVI. These results suggest that BES may serve as a potential imaging biomarker for distinguishing LVI-positive from LVI-negative breast cancer. However, studies on BES remain limited, and its clinical value requires further clarification. Future work will focus on prospective multicenter validation and AI-based analysis of breast edema subtypes to improve model accuracy and reproducibility.

## Figures and Tables

**Fig. (1):**
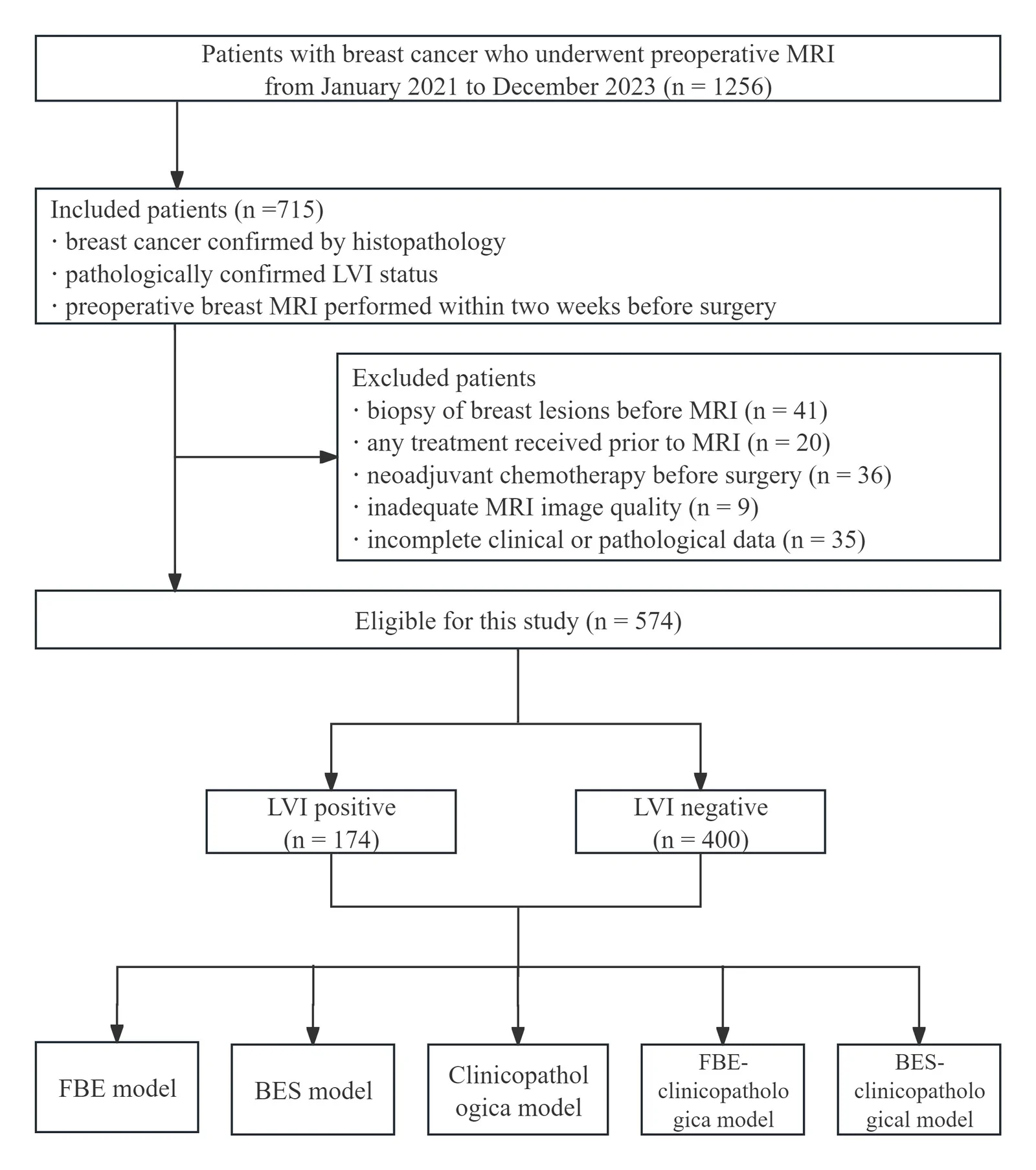
Workflow of this study.

**Fig. (2):**
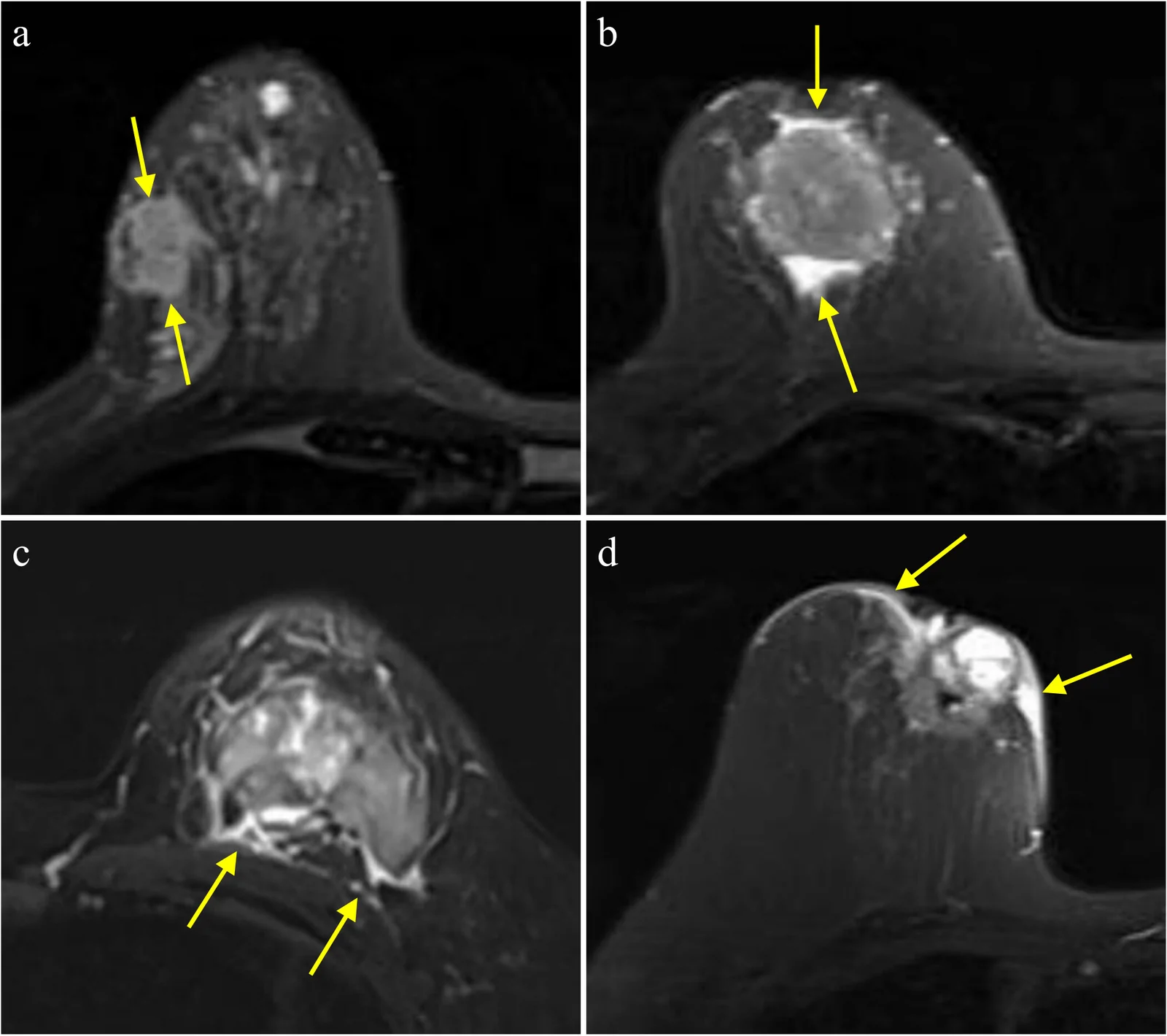
Example of each breast edema score (BES) on fat-suppressed T2-weighted imaging (T2 FS) images. (**a**) The image shows no edema (BES 1). (**b**) The image shows peritumoral edema (BES 2). (**c**) The image shows prepectoral edema (BES 3). (**d**) The image shows subcutaneous edema (BES 4).

**Fig. (3):**
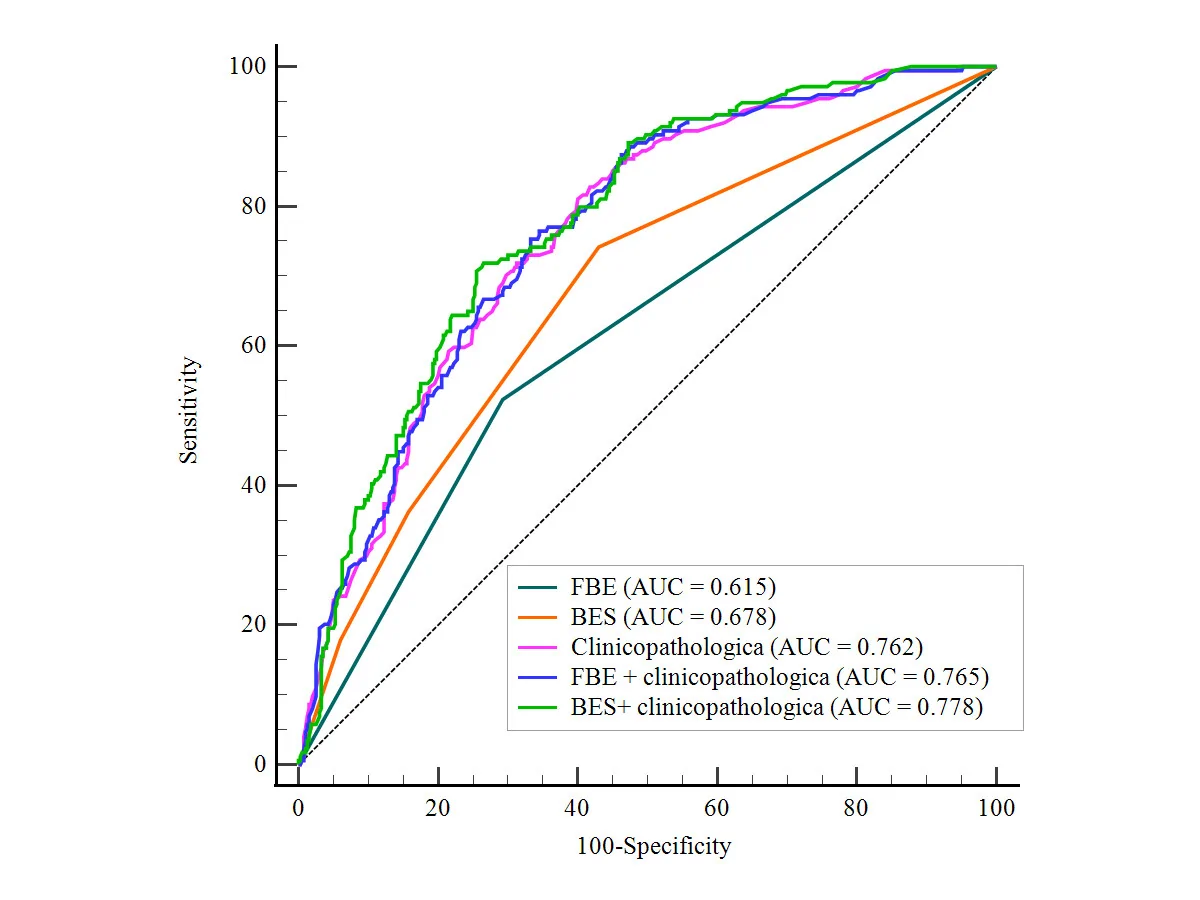
ROC curves of models for lymphovascular invasion (LVI) prediction in breast cancer.

**Table 1:** Characteristics of patients and tumors.

**Characteristics**	**LVI(n = 174)**	**Non-LVI (n = 400)**	***p*-value**
Mean age ± SD, years	55.2 ± 11.3	57.9 ± 11.0	-
Menopausal status	-	-	0.167
Premenopausal	68 (39.1%)	112 (28.0%)	-
Postmenopausal	106 (60.9%)	228 (72.0%)	-
Family history of breast cancer	-	-	0.093
No	166 (95.4%)	393 (98.2%)	-
Yes	8 (4.6%)	7 (1.8%)	-
Histologic type	-	-	0.998
Invasive ductal carcinoma	144 (82.8%)	331 (82.7%)	-
Mix/others	30 (17.2%)	69 (17.3%)	-
Axillary lymph node	-	-	0.000
Negative	75 (43.1%)	282 (70.5%)	-
Positive	99 (56.9%)	118 (29.5%)	-
Molecular subtype	-	-	0.004
Luminal A	12 (6.9%)	71 (17.7%)	-
Luminal B	125 (71.8%)	235 (58.8%)	-
HER2-positive	19 (10.9%)	46 (11.5%)	-
Triple-negative	18 (10.4%)	48 (12.0%)	-
Ki-67 index (%)	-	-	0.001
< 14	12 (6.9%)	70 (17.5%)	-
≥ 14	162 (93.1%)	330 (82.5%)	-
HER-2 status	-	-	0.376
Negative	128 (73.6%)	308 (77.0%)	-
Positive	46 (26.4%)	92 (23.0%)	-
FGT	-	-	0.002
Predominantly fatty	8 (4.6%)	19 (4.7%)	-
Scattered	33 (19.0%)	125 (31.3%)	-
Heterogeneously dense	91 (52.3%)	144 (36.0%)	-
Extremely dense	42 (24.1%)	112 (28.0%)	-
BPE	-	-	0.859
Minimal	128 (73.6%)	307 (76.7%)	-
Mild	28 (16.1%)	59 (14.8%)	-
Moderate	14 (8.0%)	26 (6.5%)	-
Marked	4 (2.3%)	8 (2.0%)	-
Lesion location	-	-	0.488
Right	89 (51.1%)	192 (48.0%)	-
Left	85 (48.9%)	208 (52.0%)	-
Maximum tumor diameter, cm	-	-	0.000
≤ 2	52 (29.9%)	279 (69.7%)	-
> 2	122 (70.1%)	121 (30.3%)	-
Lesion type	-	-	0.181
Mass	117 (67.2%)	291 (72.7%)	-
Non-mass	57 (32.8%)	109 (27.3%)	-
Mass shape	-	-	0.723
Round or oval	21 (17.9%)	48 (16.5%)	-
Irregular	96 (82.1%)	243 (83.5%)	-
Mass margin	-	-	0.089
Circumscribed	16 (13.7%)	61 (21.0%)	-
Not circumscribed	101 (86.3%)	230 (79.0%)	-
Internal enhancement pattern of the mass	-	-	0.282
Homogeneous	25 (21.4%)	79 (27.1%)	-
Heterogeneous	84 (71.8%)	185 (63.6%)	-
Rim enhancement	8 (6.8%)	27 (9.3%)	-
TIC type	-	-	0.370
Persistent type	1 (0.6%)	6 (1.5%)	-
Plateau type	103 (59.2%)	252 (63.0%)	-
Washout type	70 (40.2%)	142 (35.5%)	-
FBE	-	-	0.000
Negative	45 (25.9%)	228 (57.0%)	-
Positive	129 (74.1%)	172 (43.0%)	-
BES	-	-	0.000
1	45 (25.9%)	228 (57.0%)	-
2	66 (37.9%)	109 (27.2%)	-
3	32 (18.4%)	39 (9.8%)	-
4	31 (17.8%)	24 (6.0%)	-

**Abbreviation:** BES = breast edema score; BPE = background parenchymal enhancement; FBE = focal breast edema; FGT = fibroglandular tissue; HER2 = human epidermal growth factor receptor 2; LVI = lymphovascular invasion; SD = standard deviation; TIC = time-intensity curve.

**Table 2:** Univariate and multivariate logistic regression analyses for predictive factors of LVI.

**Variables**	**Univariate logistic**		**Multivariate logistic 1**		**Multivariate logistic 2**	
	**OR (95% CI)**	***p*-value**	**OR (95% CI)**	***p*-value**	**OR (95% CI)**	***p*-value**
Mean age ± SD, years	1.012 (0.691-4.667)	0.008	0.974 (0.956-0.992)	0.005	0.973 (0.956-0.992)	0.004
Menopausal status	0.766 (0.524-1.119)	0.168	-	-	-	-
Premenopausal	-	-	-	-	-	-
Postmenopausal	-	-	-	-	-	-
Family history of breast cancer	2.706 (0.965-7.582)	0.058	-	-	-	-
No	-	-	-	-	-	-
Yes	-	-	-	-	-	-
Histologic type	0.999 (0.624-1.601)	0.998	-	-	-	-
Invasive ductal carcinoma	-	-	-	-	-	-
Mix/others	-	-	-	-	-	-
Axillary lymph node	3.155 (2.181-4.563)	0.000	2.260 (1.431-3.568)	0.000	2.316 (1.455-3.685)	0.000
Negative	-	-	-	-	-	-
Positive	-	-	-	-	-	-
Molecular subtype	-	-	-	-	-	-
Luminal A	Ref.	-	Ref.	-	Ref.	-
Luminal B	3.147 (1.644-6.023)	0.001	3.221 (0.752-13.800)	0.115	2.859 (0.664-12.315)	0.158
HER2-positive	2.444 (1.085-5.506)	0.031	1.444 (0.315-6.612)	0.636	1.201 (0.260-5.551)	0.815
Triple-negative	2.219 (0.980-5.023)	0.056	1.248 (0.263-5.928)	0.780	1.028 (0.213-4.952)	0.973
Ki-67 index (%)	2.864 (1.509-5.434)	0.001	0.555 (0.128-2.411)	0.433	0.641 (0.145-2.824)	0.556
< 14	-	-	-	-	-	-
≥ 14	-	-	-	-	-	-
HER-2 status	1.203 (0.799-1.812)	0.376	-	-	-	-
Negative	-	-	-	-	-	-
Positive	-	-	-	-	-	-
FGT	-	-	-	-	-	-
Predominantly fatty	Ref.	-	-	-	-	-
Scattered	0.627 (0.252-1.559)	0.315	-	-	-	-
Heterogeneously dense	1.501 (0.631-3.571)	0.359	-	-	-	-
Extremely dense	0.891 (0.362-2.188)	0.801	-	-	-	-
BPE	-	-	-	-	-	-
Minimal	Ref.	-	-	-	-	-
Mild	1.138 (0.694-1.867)	0.608	-	-	-	-
Moderate	1.291 (0.653-2.553)	0.462	-	-	-	-
Marked	1.199 (0.355-4.053)	0.770	-	-	-	-
Lesion location	0.882 (0.617-1.259)	0.488	-	-	-	-
Right	-	-	-	-	-	-
Left	-	-	-	-	-	-
Maximum tumor diameter, cm	5.410 (3.668-7.978)	0.000	3.655 (2.370-5.636)	0.000	3.618 (2.337-5.601)	0.000
≤ 2	-	-	-	-	-	-
> 2	-	-	-	-	-	-
Lesion type	1.301 (0.885-1.913)	0.182	-	-	-	-
Mass	-	-	-	-	-	-
Non-mass	-	-	-	-	-	-
Mass shape	0.903 (0.513-1.588)	0.723	-	-	-	-
Round or oval	-	-	-	-	-	-
Irregular	-	-	-	-	-	-
Mass margin	1.674 (0.920-3.045)	0.091	-	-	-	-
Circumscribed	-	-	-	-	-	-
Not circumscribed	-	-	-	-	-	-
Internal enhancement pattern of the mass	-	-	-	-	-	-
Homogeneous	Ref.	-	-	-	-	-
Heterogeneous	1.435 (0.854-2.410)	0.172	-	-	-	-
Rim enhancement	0.936 (0.378-2.322)	0.887	-	-	-	-
TIC type	-	-	-	-	-	-
Persistent type	Ref.	-	-	-	-	-
Plateau type	2.452 (0.292-20.624)	0.409	-	-	-	-
Washout type	2.958 (0.349-25.045)	0.320	-	-	-	-
FBE	3.800 (2.566-5.629)	0.000	2.193 (1.366-3.522)	0.001	-	-
Negative	-	-	-	-	-	-
Positive	-	-	-	-	-	-
BES	-	-	-	-	-	-
1	Ref.	-	-	-	Ref.	-
2	3.068 (1.971-4.775)	0.000	-	-	1.825 (1.085-3.069)	0.023
3	4.157 (2.360-7.324)	0.000	-	-	2.047 (1.051-3.987)	0.035
4	6.544 (3.515-12.183)	0.000	-	-	4.341 (2.128-8.856)	0.000

**Abbreviation:** BES = breast edema score; BPE = background parenchymal enhancement; CI = confidence interval; FBE = focal breast edema; FGT = fibroglandular tissue; HER2 = human epidermal growth factor receptor 2; LVI = lymphovascular invasion; OR = odds ratio; SD = standard deviation; TIC = time-intensity curve; Multivariate logistic 1 = FBE and clinicopathological variables were included; Multivariate logistic 2 = BES and clinicopathological variables were included.

**Table 3:** Performance comparison of models for LVI prediction in breast cancer.

**Model**	**AUC (95% CI)**	**Sensitivity (%)**	**Specificity (%)**
FBE	0.615 (0.574-0.655)	52.3	70.8
BES	0.678 (0.638-0.716)	74.1	57.0
Clinicopathological	0.762 (0.725-0.796)	81.0	60.0
FBE+ clinicopathological	0.765 (0.728-0.799)	75.3	66.7
BES+ clinicopathological	0.778 (0.742-0.811)	71.8	73.5

**Abbreviation:** AUC = area under curve; BES = breast edema score; CI = confidence interval; FBE = focal breast edema; LVI = lymphovascular invasion.

## Data Availability

The data that support the findings of this study are available from the corresponding authors, [X.W.] and [H.Y.L.], upon reasonable request.

## LIST OF ABBREVIATIONS

ACR: American College of Radiology
ADC: Apparent Diffusion Coefficient
AI: Artificial Intelligence
AUC: Area Under the ROC Curve
ALN: Axillary Lymph Node
BES: Breast Edema Score
BI-RADS: Breast Imaging Reporting and Data System
BPE: Background Parenchymal Enhancement
CI: Confidence Interval
DCE: Dynamic Contrast-enhanced
ER: Estrogen Receptor
FBE: Focal Breast Edema
FGT: Fibroglandular Tissue
FISH: Fluorescence in Situ Hybridization
FOV: Field of View
HER2: Human Epidermal Growth Factor Receptor 2
IDC: Invasive Ductal Carcinoma
LVI: Lymphovascular Invasion
MRI: Magnetic Resonance Imaging
OR: Odds Ratio
PR: Progesterone Receptor
ROC: Receiver Operating Characteristic
RSNA: Radiological Society of North America
SBI: Society of Breast Imaging
T1WI: T1-weighted Imaging
T2WI: T2-weighted Imaging
FS: Fat-suppressed
TE: Echo Time
TIC: Time-signal Intensity Curve
TR: Repetition Time

## STANDARDS OF REPORTING

STROBE guidelines were followed.

## References

[1] Sung H., Ferlay J., Siegel R.L., Laversanne M., Soerjomataram I., Jemal A., Bray F. (2021). Global cancer statistics 2020: GLOBOCAN estimates of incidence and mortality worldwide for 36 cancers in 185 countries.. CA Cancer J. Clin..

[2] Liu Y.L., Saraf A., Lee S.M., Zhong X., Hibshoosh H., Kalinsky K., Connolly E.P. (2016). Lymphovascular invasion is an independent predictor of survival in breast cancer after neoadjuvant chemotherapy.. Breast Cancer Res. Treat..

[3] Ahn K.J., Park J., Choi Y. (2017). Lymphovascular invasion as a negative prognostic factor for triple-negative breast cancer after surgery.. Radiat. Oncol. J..

[4] Akrami M., Meshksar A., Ghoddusi J.M., Safarpour M.M., Tahmasebi S., Zangouri V., Talei A. (2021). Prognostic role of lymphovascular invasion in patients with early breast cancer.. Indian J. Surg. Oncol..

[5] Zhong Y.M., Tong F., Shen J. (2022). Lympho-vascular invasion impacts the prognosis in breast-conserving surgery: A systematic review and meta-analysis.. BMC Cancer.

[6] Cheon H., Kim H.J., Lee S.M., Cho S.H., Shin K.M., Kim G.C., Park J.Y., Kim W.H. (2017). Preoperative MRI features associated with lymphovascular invasion in node-negative invasive breast cancer: A propensity-matched analysis.. J. Magn. Reson. Imaging.

[7] Ouyang F., Guo B., Huang X., Ouyang L., Zhou C., Zhang R., Wu M., Yang Z., Wu S., Guo T., Yang S., Hu Q. (2019). A nomogram for individual prediction of vascular invasion in primary breast cancer.. Eur. J. Radiol..

[8] Uematsu T. (2015). Focal breast edema associated with malignancy on T2-weighted images of breast MRI: Peritumoral edema, prepectoral edema, and subcutaneous edema.. Breast Cancer.

[9] Harada T.L., Uematsu T., Nakashima K., Kawabata T., Nishimura S., Takahashi K., Tadokoro Y., Hayashi T., Tsuchiya K., Watanabe J., Sugino T. (2021). Evaluation of breast edema findings at T2-weighted breast MRI is useful for diagnosing occult inflammatory breast cancer and can predict prognosis after neoadjuvant chemotherapy.. Radiology.

[10] Xie S., Tang W., Zhang C., Wang J., Wang M., Zhou Y. (2024). Classification of breast edema on T2-weighted imaging for predicting sentinel lymph node metastasis and biological behavior in breast cancer.. Clin. Radiol..

[11] Deniz Altıntaş D., Esen Icten G., Taşkın F., Uras C. (2025). Relationships between breast edema and axillary lymph node metastasis in breast cancer.. Diagnostics.

[12] Xie T., Huang Y., Xiao Q., Peng W., Zhao Q., Gu Y. (2025). Preoperative focal breast edema on T2-weighted MRI as a predictor of sentinel lymph node metastasis in clinically node-negative T1-2 breast cancer: A retrospective bicentric study.. Eur. J. Radiol..

[13] Zhu Y., Zhang S., Wei W., Yang L., Wang L., Wang Y., Fan Y., Sun H., Xie Z. (2025). Intra- and peritumoral radiomics nomogram based on DCE-MRI for the early prediction of pathological complete response to neoadjuvant chemotherapy in breast cancer.. Front. Oncol..

[14] Yilmaz R., Comert R.G., Bayram A., Cingoz E., Onder S., Karli E.H., Muslumanoglu M. (2025). Role of breast edema in predicting complete response to neoadjuvant chemotherapy in clinical axillary-positive breast cancer.. Jpn. J. Radiol..

[15] Wolff A.C., Hammond M.E.H., Allison K.H., Harvey B.E., Mangu P.B., Bartlett J.M.S., Bilous M., Ellis I.O., Fitzgibbons P., Hanna W., Jenkins R.B., Press M.F., Spears P.A., Vance G.H., Viale G., McShane L.M., Dowsett M. (2018). Human epidermal growth factor receptor 2 testing in breast cancer: American Society of clinical oncology/college of American pathologists clinical practice guideline focused update.. J. Clin. Oncol..

[16] Goldhirsch A., Winer E.P., Coates A.S., Gelber R.D., Piccart-Gebhart M., Thürlimann B., Senn H.J., Albain K.S., André F., Bergh J., Bonnefoi H., Bretel-Morales D., Burstein H., Cardoso F., Castiglione-Gertsch M., Coates A.S., Colleoni M., Costa A., Curigliano G., Davidson N.E., Di Leo A., Ejlertsen B., Forbes J.F., Gelber R.D., Gnant M., Goldhirsch A., Goodwin P., Goss P.E., Harris J.R., Hayes D.F., Hudis C.A., Ingle J.N., Jassem J., Jiang Z., Karlsson P., Loibl S., Morrow M., Namer M., Kent Osborne C., Partridge A.H., Penault-Llorca F., Perou C.M., Piccart-Gebhart M.J., Pritchard K.I., Rutgers E.J.T., Sedlmayer F., Semiglazov V., Shao Z-M., Smith I., Thürlimann B., Toi M., Tutt A., Untch M., Viale G., Watanabe T., Wilcken N., Winer E.P., Wood W.C., Panel members (2013). Personalizing the treatment of women with early breast cancer: highlights of the St Gallen International Expert Consensus on the Primary Therapy of Early Breast Cancer 2013.. Ann. Oncol..

[17] Xu Z., Ding Y., Zhao K., Han C., Shi Z., Cui Y., Liu C., Lin H., Pan X., Li P., Chen M., Wang H., Deng X., Liang C., Xie Y., Liu Z. (2022). MRI characteristics of breast edema for assessing axillary lymph node burden in early-stage breast cancer: a retrospective bicentric study.. Eur. Radiol..

[18] Uematsu T., Kasami M., Watanabe J. (2014). Is evaluation of the presence of prepectoral edema on T2-weighted with fat-suppression 3 T breast MRI a simple and readily available noninvasive technique for estimation of prognosis in patients with breast cancer?. Breast Cancer.

[19] Zhou P., Jin C., Lu J., Xu L., Zhu X., Lian Q., Gong X. (2021). The Value of Nomograms in Pre-Operative Prediction of Lymphovascular Invasion in Primary Breast Cancer Undergoing Modified Radical Surgery: Based on Multiparametric Ultrasound and Clinicopathologic Indicators.. Ultrasound Med. Biol..

[20] Zhang C., Liang Z., Feng Y., Xiong Y., Manwa C., Zhou Q. (2023). Risk Factors for Lymphovascular Invasion in Invasive Ductal Carcinoma Based on Clinical and Preoperative Breast MRI Features: a Retrospective Study.. Acad. Radiol..

[21] Houvenaeghel G., Cohen M., Classe J.M., Reyal F., Mazouni C., Chopin N., Martinez A., Daraï E., Coutant C., Colombo P.E., Gimbergues P., Chauvet M.P., Azuar A.S., Rouzier R., Tunon de Lara C., Muracciole X., Agostini A., Bannier M., Charaffe Jauffret E., De Nonneville A., Goncalves A. (2021). Lymphovascular invasion has a significant prognostic impact in patients with early breast cancer, results from a large, national, multicenter, retrospective cohort study.. ESMO Open.

[22] Chollet-Hinton L., Anders C.K., Tse C.K., Bell M.B., Yang Y.C., Carey L.A., Olshan A.F., Troester M.A. (2016). Breast cancer biologic and etiologic heterogeneity by young age and menopausal status in the Carolina Breast Cancer Study: a case-control study.. Breast Cancer Res..

[23] Jacquemier J., Charafe-Jauffret E., Monville F., Esterni B., Extra J.M., Houvenaeghel G., Xerri L., Bertucci F., Birnbaum D. (2009). Association of GATA3, P53, Ki67 status and vascular peritumoral invasion are strongly prognostic in luminal breast cancer.. Breast Cancer Res..

[24] Shen S., Wu G., Xiao G., Du R., Hu N., Xia X., Zhou H. (2018). Prediction model of lymphovascular invasion based on clinicopathological factors in Chinese patients with invasive breast cancer.. Medicine (Baltimore).

[25] Ugras S., Stempel M., Patil S., Morrow M. (2014). Estrogen receptor, progesterone receptor, and HER2 status predict lymphovascular invasion and lymph node involvement.. Ann. Surg. Oncol..

[26] Zhang S., Zhang D., Yi S., Gong M., Lu C., Cai Y., Tang X., Zou L. (2017). The relationship of lymphatic vessel density, lymphovascular invasion, and lymph node metastasis in breast cancer: a systematic review and meta-analysis.. Oncotarget.

[27] Liu Z., Feng B., Li C., Chen Y., Chen Q., Li X., Guan J., Chen X., Cui E., Li R., Li Z., Long W. (2019). Preoperative prediction of lymphovascular invasion in invasive breast cancer with dynamic contrast-enhanced-MRI-based radiomics.. J. Magn. Reson. Imaging.

[28] Coşkun BA, Yaltırık BE, Bulut ZM, Esen BI, Bilgin E (2024). Preoperative dynamic contrast-enhanced and diffusion-weighted breast magnetic resonance imaging findings for prediction of lymphovascular invasion of the lesions in node-negative invasive breast cancer.. Can Assoc Radiol J.

[29] Choi W.J., Cha J.H., Kim H.H., Shin H.J., Chae E.Y. (2016). The accuracy of breast MR imaging for measuring the size of a breast cancer: Analysis of the histopathologic factors.. Clin. Breast Cancer.

[30] Kim J.Y., Kim S.H., Kim Y.J., Kang B.J., An Y.Y., Lee A.W., Song B.J., Park Y.S., Lee H.B. (2015). Enhancement parameters on dynamic contrast enhanced breast MRI: do they correlate with prognostic factors and subtypes of breast cancers?. Magn. Reson. Imaging.

[31] Li J., Mo Y., He B., Gao Q., Luo C., Peng C., Zhao W., Ma Y., Yang Y. (2019). Association between MRI background parenchymal enhancement and lymphovascular invasion and estrogen receptor status in invasive breast cancer.. Br. J. Radiol..

[32] Sun R, Hu Y, Wang X (2024). Intratumoral and peritumoral edema radiomics based on fat-suppressed T2- weighted imaging for preoperative prediction of triple-negative breast cancer.. Curr Med Imaging..

[33] Zhao S., Li Y., Ning N., Liang H., Wu Y., Wu Q., Wang Z., Tian J., Yang J., Gao X., Liu A., Song Q., Zhang L. (2024). Association of peritumoral region features assessed on breast MRI and prognosis of breast cancer: a systematic review and meta-analysis.. Eur. Radiol..

[34] Harada T.L., Uematsu T., Nakashima K., Sugino T., Nishimura S., Takahashi K., Hayashi T., Tadokoro Y., Watanabe J., Nakamoto S., Ito T. (2020). Is the presence of edema and necrosis on T2WI pretreatment breast MRI the key to predict pCR of triple negative breast cancer?. Eur. Radiol..

[35] Mohammed R.A.A., Martin S.G., Mahmmod A.M., Macmillan R.D., Green A.R., Paish E.C., Ellis I.O. (2011). Objective assessment of lymphatic and blood vascular invasion in lymph node-negative breast carcinoma: findings from a large case series with long-term follow-up.. J. Pathol..

